# Evaluating the survival outcomes in clinical node stage 2 and 3 breast cancer patients with negative sentinel lymph node biopsy after neoadjuvant chemotherapy: sentinel lymph node biopsy alone vs. axillary lymph node dissection

**DOI:** 10.3389/fonc.2025.1563586

**Published:** 2025-05-20

**Authors:** Eunju Shin, Tae-Kyung Yoo, Jisun Kim, Il Yong Chung, Beom Seok Ko, Hee Jeong Kim, Jong Won Lee, Byung Ho Son, Sae Byul Lee

**Affiliations:** Division of Breast Surgery, Department of Surgery, University of Ulsan College of Medicine, Asan Medical Center, Seoul, Republic of Korea

**Keywords:** breast cancer, neoadjuvant chemotherapy, axillary surgery, sentinel lymph node biopsy, axillary lymph node dissection

## Abstract

**Purpose:**

With the advancement of neoadjuvant chemotherapy (NAC), the reliance on surgical removal of axillary for high-risk breast cancer is diminishing. However, there is a lack of data on the oncologic safety of sentinel lymph node biopsy (SNB) alone in patients with clinical node stages 2 and 3 who show a favorable response to NAC. This study aims to compare the oncologic outcomes of SNB alone versus SNB combined with axillary lymph node dissection (ALND) in this patient cohort.

**Methods:**

Conducted at Asan Medical Center, this retrospective study analyzed data from breast cancer patients treated with NAC between 2008 and 2021. Propensity score matching (PSM) was employed to compare patients based on treatment approach. SNB was performed on patients demonstrating significant response to NAC with minimal nodal involvement and ALND was reserved for cases with negative SNB results, as determined by the operating surgeon. The study evaluated oncologic safety by comparing axillary recurrence-free survival (ARFS), regional recurrence-free survival (RRFS), and overall survival (OS) across surgical methods.

**Results:**

Over a median follow-up of 44 months, the overall axillary recurrence rate was 2.3%, and the univariate and multivariate analyses showed no statistically significant differences in ARFS, RRFS, and OS between the groups. Propensity score-matched analysis further confirmed the absence of significant differences in 5-year ARFS, RRFS, and OS outcomes between the SNB-only and ALND groups.

**Conclusions:**

This study demonstrates that performing sentinel node biopsy alone is feasible in patients with clinical node stage 2–3 after neoadjuvant chemotherapy. The findings suggest the potential for de-escalation of axillary management in these patients, which could be further explored in follow-up studies.

## Introduction

Axillary management in breast cancer has been advanced over the years, from extensive procedures such as axillary lymph node dissection (ALND) to minimally invasive techniques like sentinel lymph node biopsy (SNB) and targeted axillary dissection (TAD). In the area of surgery, the shift towards surgical de-escalation aims to reduce the physical burdens of axillary interventions while enhancing the effectiveness of complementary treatments. This is reflected in ongoing clinical trials, such as SOUND, INSEMA, and NAUTILUS in the upfront surgery, and NSABP B-51 and Alliance A011202 in the neoadjuvant setting (1–5). These studies aims to shift towards minimizing surgical aggressiveness- to decrease complications and enriching patient quality of life. But still, these developments have not only fueled considerable academic debate but have also sparked an inquiries concerning their safety and efficacy.

Recent studies have focused on comparing LNB alone versus SNB plus ALND in patients with axillary metastasis before neoadjuvant chemotherapy. Several studies generally indicate that both procedures have no difference in outcomes, suggesting that SNB alone may OK in this specific cohort (6–8). However, the literature presents heterogeneous findings; while some studies report equivalent clinical results between the two groups, others show the necessity for additional axillary management in certain patient populations. And these findings are predominantly about patients classified at clinical node stage 1.

These leads to a pertinent question concerning higher stage of nodal metastasis, such as clinical node stage 2 and 3. With advancement in neoadjuvant chemotherapy enhancing treatment outcomes, it becomes crucial to examine these higher stages more closely. Our research seeks to shed light on the management of clinical node stage 2 or 3 breast cancer patients after neoadjuvant chemotherapy, particularly when SNB results are negative at the time of surgery. The objective of our study is not sole to document retrospective outcomes but also to align surgical practices with the existing evidence, advocating for the necessity of prospective studies. Such efforts are aimed at optimizing the treatment paradigm in breast cancer surgery.

## Methods

### Study population

**Figure 1:**
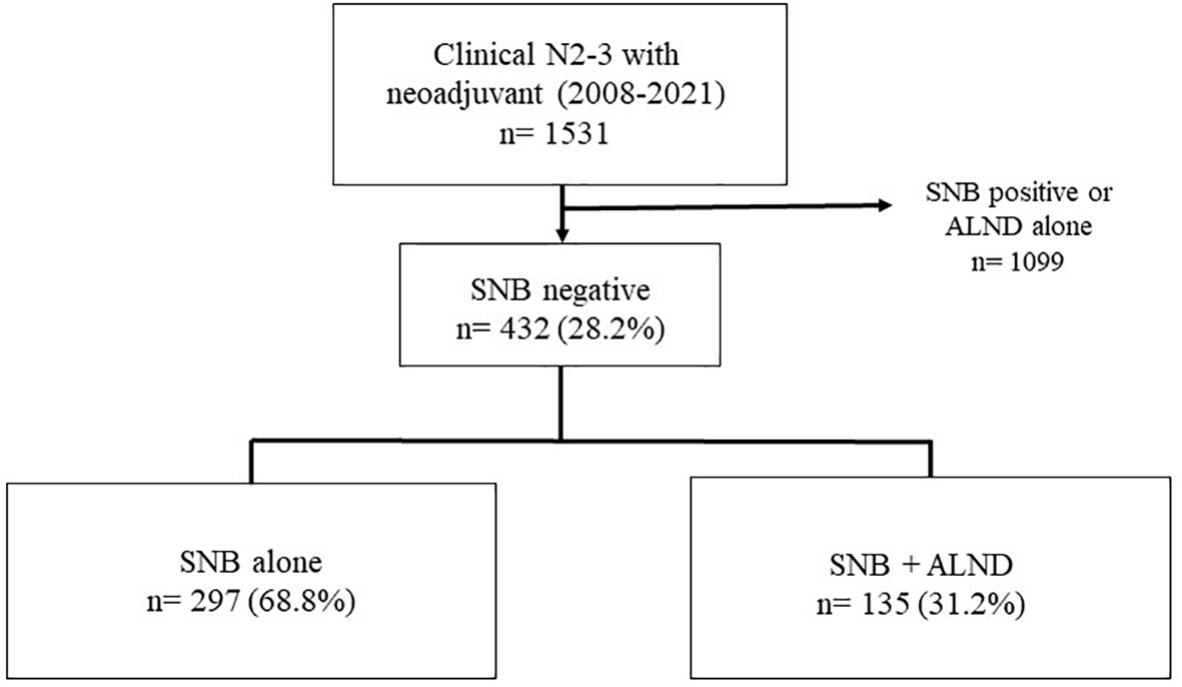
Study population.

This retrospective study of patients who underwent surgery after neoadjuvant chemotherapy at Asan Medical Center from 2008 to 2021. The initial cohort comprised 1,531 patients who underwent surgical treatment post-NAC for breast cancer at clinical node stages 2 and 3 during the specified period. Patients who tested positive for sentinel lymph nodes (SNB) or underwent axillary lymph node dissection (ALND) without prior SNB were excluded from further analysis. These exclusion criteria refined the cohort to 432 eligible patients. Among these, 297 patients underwent SNB alone, and 135 underwent SNB followed by ALND. **Figure 1** illustrates the distribution and selection process of the study population.

This study was reviewed and approved by the Institutional Review Board of the Asan Medical Center (2022-0282) and informed consent was waived because the study was based on retrospective clinical data. And data were accessed for research purposes from January to February 2023.

### Surgical methods

The SNB was performed using a dual approach involving both radioisotope and blue dye to ensure accurate sentinel node identification. In cases where axillary nodes were clinically enlarged or palpable, these nodes were also excised during the procedure. Previously, for patients with clinically node-positive stage 2 or 3 before neoadjuvant chemotherapy, performing an ALND was the standard approach. However, in patients whose SNB results were negative at the time of surgery, for whom imaging before and after neoadjuvant chemotherapy suggested a good therapeutic response, and with no evidence of lymph node enlargement, the axillary surgery was completed with SNB alone, without proceeding to ALND.

### Outcome measures

The primary endpoints of this study included axillary recurrence-free survival (ARFS), regional recurrence-free survival (RRFS), Disease-free survival (DFS) and overall survival (OS). ARFS is the time from the date of surgery to another axillary recurrence including recurrent carcinoma *in situ*, RRFS is defined as a metastatic disease in the ipsilateral axillary, internal mammary or supraclavicular or infraclavicular nodes, with or without involvement of the ipsilateral breast tissue. OS was defined as the time from the date of diagnosis of breast cancer to any deaths, whether they were breast cancer-related or not.

These metrics were chosen to evaluate the effectiveness of the surgical intervention in preventing recurrence and prolonging life in the patient population under study.

### Statistical analysis

We analyzed the baseline variables, which were stratified by the axillary surgery methods, by performing two-sided chi-squared analysis, Fisher’s exact test, and Mann–Whitney U test to determine the significance of the results. ARFS, RRFS and OS were plotted using the Kaplan–Meier product-limit method, and log-rank p-value was calculated. To assess the prognostic impact of the clinicopathologic factors, hazard ratios, 95% confidence intervals, and p-value were calculated using the Cox proportional hazards model. All statistical tests were two-tailed, and a p-value less than 0.05 was considered statistically significant. Statistical analyses were performed using the Statistical Package for the Social Sciences (SPSS, ver. 20, Armonk, NY, USA).

## Results

### Patients’ characteristics

The median follow-up for this study was 44 months, and the overall axillary recurrence rate was 2.3%. A total of 432 patients were analyzed, including 297 in the SNB only group and 135 in the SNB + ALND group. Age distribution and initial clinical node stages did not significantly differ between the groups (*p*=0.756 and *p*=0.513, respectively). However, initial clinical T stage displayed some variation, with a slightly higher presence of T0 and T4 stages in the SNB + ALND group, which was statistically significant (*p*=0.041). The number of metastatic nodes showed a significant difference, with no metastatic nodes found in all patients of the SNB only group, whereas some patients in the SNB + ALND group had up to 4 or more metastatic nodes (*p*=0.001). Additionally, in the SNB+ALND groups, there were 6 cases of metastatic nodes, 5 of which had over 4 metastatic nodes (**Table 1**).

**Table 1:** Baseline characteristics.

	Total	SLNB only N= 297 (%)	SNB + ALND N= 135 (%)	*p*-value
**Age at diagnosis (years)**				0.756
50 ≥	228 (52.8)	155 (52.2)	73 (54.1)	
50 <	204 (47.2)	142 (47.8)	62 (45.9)	
**Initial clinical T stage**				0.041
0	5 (1.2)	1 (0.3)	4 (3.0)	
1	39 (9.0)	32 (10.8)	7 (5.2)	
2	234 (54.2)	162 (54.5)	72 (53.3)	
3	113 (26.2)	77 (25.9)	36 (26.7)	
4	41 (9.5)	25 (8.4)	16 (11.9)	
**Initial clinical N stage**				0.513
2	148 (34.3)	105 (35.4)	43 (31.9)	
3	284 (65.7)	192 (64.6)	92 (68.1)	
**Pathologic T stage**				0.917
CR	251 (58.1)	172 (57.9)	79 (58.5)	
Non-CR	181 (41.9)	125 (42.1)	56 (41.5)	
**Number of metastatic node(s)**				0.001
0	426 (98.6)	297 (100.0)	129 (95.6)	
1-3	1 (0.2)	0 (0.0)	1 (0.7)	
4 ≥	5 (1.2)	0 (0.0)	5 (3.7)	
**Breast surgery**				0.215
Breast conserving surgery	221 (51.2)	158 (53.2)	63 (46.7)	
Mastectomy	211 (48.8)	139 (46.8)	72 (53.3)	
**Histologic grade**				0.145
2	221 (51.5)	160 (53.9)	61 (46.2)	
3	208 (48.5)	137 (46.1)	71 (53.8)	
Unknown	3	0	3	
**Nuclear grade**				0.210
2	218 (50.9)	157 (53.0)	61 (46.2)	
3	210 (49.1)	139 (47.0)	71 (53.8)	
Unknown	4	1	3	
**LVI**				0.611
Absent	237 (86.4)	166 (85.6)	101 (87.8)	
Present	42 (13.6)	28 (14.4)	14 (12.2)	
Unknown	123	103	20	
**ER**				0.835
Negative	241 (55.8)	167 (56.2)	74 (54.8)	
Positive	191 (44.2)	130 (43.8)	61 (45.2)	
**PR**				0.261
Negative	337 (78.0)	227 (76.4)	110 (81.5)	
Positive	95 (22.0)	70 (23.6)	25 (18.5)	
**HER2 status**				0.678
Negative	212 (49.1)	148 (49.8)	64 (47.4)	
Positive	220 (50.9)	149 (50.2)	71 (52.6)	
**Ki-67**				1.000
20 ≥	403 (96.0)	278 (95.9)	125 (96.2)	
20 <	17 (4.0)	12 (4.1)	5 (3.8)	
Unknown	12	7	5	
**Radiation therapy**				0.204
No	19 (4.4)	16 (5.4)	3 (2.2)	
Yes	413 (95.6)	281 (94.6)	132 (97.8)	
**Endocrine therapy**				0.835
No	239 (55.3)	163 (54.9)	76 (56.3)	
Yes	193 (44.7)	134 (45.1)	59 (43.7)	

**Table 2** shows the comparison of patient characteristics after propensity score matching, maintaining a balanced distribution between the two groups, each consisting of 124 patients. Both groups showed similar distributions in terms of age, initial clinical T and N stages, and other pathological features, indicating successful matching.

**Table 2:** Number of sentinel lymph node obtained.

	SNB only N (%)	SNB +ALND N (%)	P -value
**Number of sentinel nodes**			0.000
1	14 (4.7)	16 (11.9)	
2	25 (8.4)	30 (22.2)	
3	57 (19.2)	26 (19.3)	
4	51 (17.2)	26 (19.3)	
5	122 (41.1)	27 (20.0)	
>5	28 (9.4)	10 (7.4)	
**Mean (standard deviation)**	5.0 (1.36)	3.0 (1.51)	
**Largest invasion depth (mm)**	0	20	

### Sentinel lymph node retrieval

According to **Table 3**, the number of sentinel lymph nodes retrieved varied significantly between the two groups (p<0.0001). The SNB only group had a higher mean number of sentinel nodes retrieved (5.0 ± 1.36) compared to the SNB + ALND group (3.0 ± 1.51). In the SNB+ALND group, the average largest invasion depth was 20mm.

**Table 3:** Comparison of characteristics of patients after propensity score matching.

	Total	SLNB only N=124 (%)	SNB + ALND N=124 (%)
Age at diagnosis (years)			
50 ≥	117 (47.2)	58 (46.8)	59 (47.6)
50 <	131 (52.8)	66 (53.2)	65 (52.4)
Initial clinical T stage			
0	3 (1.2)	1 (0.8)	2 (1.6)
1	17 (6.9)	10 (8.1)	7 (5.6)
2	138 (55.6)	67 (54.0)	71 (57.3)
3	67 (27.0)	32 (25.8)	35 (28.2)
4	9.3 (9.3)	14 (11.3)	9 (7.3)
Initial clinical N stage			
2	76 (30.6)	38 (30.6)	38 (30.6)
3	172 (69.4)	86 (69.4)	86 (69.4)
Pathologic T stage			
CR	97 (39.1)	48 (38.7)	49 (39.5)
Non-CR	151 (60.9)	76 (61.3)	75 (60.5)
Number of metastatic node(s)			
0	241 (97.6)	124 (100.0)	117 (95.1)
1-3	1 (0.4)	0 (0.0)	1 (0.8)
4 ≥	5 (2.0)	0 (0.0)	5 (4.1)
Breast surgery			
Breast conserving surgery	114 (46.0)	56 (42.7)	61 (49.2)
Mastectomy	134 (54.0)	71 (57.3)	63 (50.8)
**Histologic grade**			
2	126 (51.2)	70 (56.5)	56 (45.9)
3	120 (48.8)	53 (43.5)	66 (54.1)
Unknown	2	0	2
Nuclear grade			
2	125 (51.0)	70 (56.9)	55 (45.1)
3	120 (49.0)	53 (43.1)	67 (54.9)
Unknown	3	1	2
**LVI**			
Absent	163 (87.2)	68 (86.1)	95 (88.0)
Present	24 (12.8)	11 (13.9)	13 (12.0)
Unknown	61	45	16
ER			
Negative	138 (55.6)	70 (59.5)	68 (54.8)
Positive	110 (44.4)	54 (43.5)	56 (45.2)
PR			
Negative	202 (81.5)	102 (82.3)	100 (80.6)
Positive	46 (18.5)	22 (17.7)	24 (19.4)
HER2 status			
Negative	140 (49.0)	82 (50.6)	58 (46.8)
Positive	146 (51.0)	80 (49.4)	66 (53.2)
Ki-67			
20 ≥	231 (95.9)	116 (95.6)	115 (95.8)
20 <	10 (4.1)	5 (4.4)	5 (4.2)
Unknown	7	3	4
Radiation therapy			
No	12 (4.8)	10 (8.1)	2 (1.6)
Yes	236 (95.2)	114 (91.9)	122 (98.4)
Endocrine therapy			
No	140 (56.5)	70 (56.5)	70 (56.5)
Yes	108 (43.5)	54 (43.5)	54 (43.5)

### Survival outcomes according to axillary surgery

**Figure 2:**
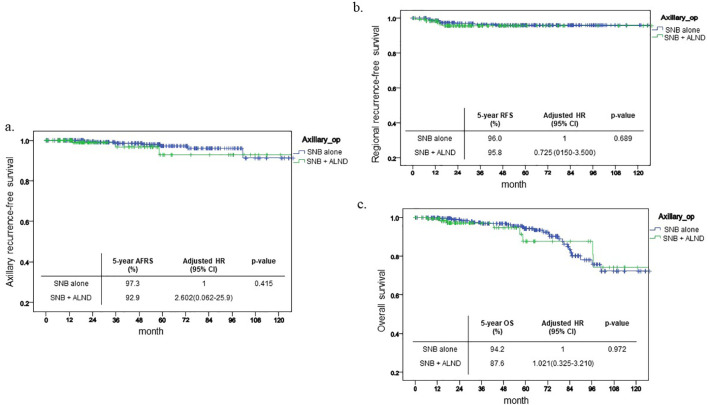
Kaplan-Meier curve before PSM. This shows Kaplan-Meier curve of each outcome before PSM and 5-year outcome and its HR. **(a)** ARFS **(b)** RFS **(c)** OS.

Prior to propensity score matching, survival analyses were conducted across the entire cohort. The five-year axillary recurrence-free survival (ARFS) rate for patients undergoing SNB alone was 97.3%, compared to 92.9% for those in the SNB+ALND group, with no statistically significant difference observed between the two groups (p=0.415, **Figure 2a**). Likewise, the five-year regional recurrence-free survival (RRFS) rates were 96% for the SNB alone group and 95.8% for the SNB+ALND group, again showing no significant disparity (p=0.689, **Figure 2b**). Regarding overall survival (OS), while a slight difference was noted—94.2% in the SNB alone group versus 87.6% in the SNB+ALND group—this difference did not reach statistical significance (p=0.972, **Figure 2c**). Additionally, multivariable analyses revealed no significant differences across all measured outcomes.

**Figure 3:**
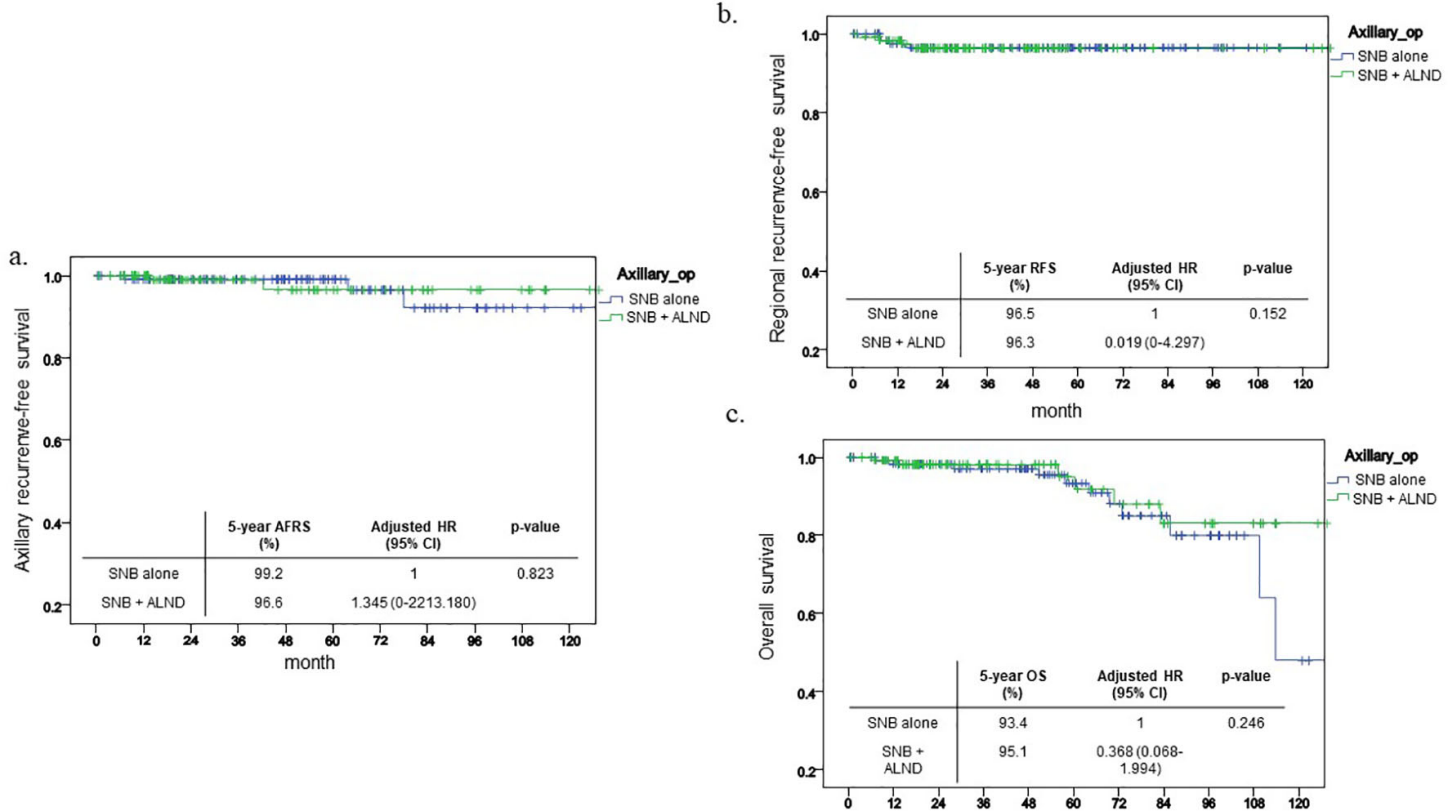
Kaplan-Meier curve after PSM. This shows Kaplan-Meier curve of each outcome after PSM and 5-year outcome and its HR. **(a)** ARFS **(b)** RFS **(c)** OS.

Following propensity score matching, the comparative results between the two groups remained statistically non-significant. The five-year axillary recurrence-free survival (ARFS) rate was 99.2% for the SNB group compared to 96.6% for the SNB+ALND group, demonstrating no statistically significant differences (p=0.823, **Figure 3a**). Similarly, no significant differences were observed in the outcomes concerning regional recurrence-free survival (RRFS) and overall survival (OS), with p-values of 0.152 and 0.246, respectively (**Figures 3b, c**).

**Table 4** presents the HR for each outcome across univariate and multivariate analyses, as well as after PSM. No significant differences were observed in ARFS, RFS, OS between SNB-only group and plus ALND group. However, in the case of DFS, a lower hazard was noted in the SNB + ALND group in the univariate analysis (HR = 0.479, *p* = 0.017), whereas a higher hazard was observed in the multivariate analysis (HR = 2.448, *p* = 0.021). Nevertheless, this difference was not statistically significant after PSM (HR = 2.258, *p* = 0.131).

**Table 4:** Cox regression.

Variables	No, of events	Univariate		Multivariate		PSM	
		HR (95% CI)	*p* -value	HR (95% CI)	*p* -value	HR (95% CI)	*p* -value
ARFS							
SNB only	7	1 (Ref)	0.473	1 (Ref)	0.415	1 (Ref)	0.968
SNB + ALND	3	0.605 (0.154-2.382)		2.602 (0.062-25.892)		1.345 (0.000-2213.180)	
RFS							
SNB only	6	1 (Ref)	0.680	1 (Ref)	0.689	1 (Ref)	0.152
SNB + ALND	4	0.797 (0.272-2.341)		0.725 (0.150-3.500)		0.019 (0.000-4.297)	
DFS							
SNB only	14	1 (Ref)	0.017	1 (Ref)	0.021	1 (Ref)	0.131
SNB + ALND	16	0.479 (0.262-0.875)		2.448 (1.141-5.250)		2.258 (0.784-6.505)	
OS							
SNB only	15	1 (Ref)	0.700	1 (Ref)	0.972	1 (Ref)	0.246
SNB + ALND	7	0.858 (0.395-1.866)		1.021 (0.325-3.210)		0.368 (0.068-1.994)	

## Discussion

In this retrospective study, we analyzed 432 breast cancer patients with clinical node stages 2 and 3 who underwent axillary surgery following neoadjuvant chemotherapy (NAC). Among them, 297 received sentinel lymph node biopsy (SNB) alone and 135 underwent SNB followed by axillary lymph node dissection (ALND). Over a median follow-up of 44 months, the overall axillary recurrence rate was 2.3%. Survival outcomes—axillary recurrence-free survival (ARFS), regional recurrence-free survival (RRFS), and overall survival (OS)—showed no significant differences between groups either before or after propensity score matching. The findings suggest that SNB alone might suffice for certain patients with clinical node stage 2 and 3 breast cancer post-NAC, without compromising oncologic outcomes.

Several landmark studies have explored the reduction of axillary surgery in patients with clinically node-positive breast cancer who have undergone neoadjuvant chemotherapy (NAC). Research such as the ACOSOG Z1071, SENTINA, and SNFNAC trials have significantly contributed to the understanding of de-escalating axillary lymph node dissection (ALND) and its associated morbidity (8).

The ACOSOG Z1071 trial reported that up to 40% of patients with node-positive disease at diagnosis achieve a pathological complete response (pCR) in the axilla following NAC (9). It also noted a false negative rate (FNR) of 12.6% to 14.2% when more than two sentinel lymph nodes were harvested, with no significant variation based on pretreatment nodal status (*p*=0.51). Another study assessing the reliability of sentinel lymph node biopsy (SNB) post-NAC found an FNR of 13% and an identification rate of 91% (10).

The SENTINA study further highlighted that the FNR decreases to 7% or less when three or more sentinel nodes are removed, compared to 19% with two nodes and 24% with only one node removed (11).

One of the primary concerns in this study, as in others investigating SNB in the post-NAC setting, is the risk of false-negative results. This can be particularly problematic in patients with advanced nodal disease. To mitigate this risk, it is crucial to ensure an adequate number of sentinel lymph nodes are retrieved during the biopsy. Current guidelines suggest that a minimum of two sentinel nodes should be excised to reduce the likelihood of false negatives, especially in patients with more advanced nodal disease. Achieving this standard is critical to ensuring the accuracy of SNB and avoiding under-treatment of axillary disease. In our study, sentinel nodes were acquired in 76.4% of patients with more than two nodes retrieved, and in 91.7% of cases where more than two nodes were removed, suggesting a prospectively low false FNR. Interestingly, the mean number of sentinel nodes removed differed between the two groups, with a higher number extracted in the SNB only group. This variation may be influenced by the surgical operator’s intent, reflecting preoperative decisions made regarding the patient’s therapeutic plan.

Numerous ongoing trials are investigating the potential for de-escalating axillary surgery in patients who demonstrate a favorable response to neoadjuvant chemotherapy (NAC). Notable among these is the AXSANA (AXillary Surgery After NeoAdjuvant Treatment) trial (12), a prospective multicenter cohort study conducted by EUBREAST, and the MINIMAX (MINImal versus MAXimal Invasive Axillary Staging and Treatment After Neoadjuvant Systemic Therapy in Node Positive Breast Cancer) (13), a Dutch multicenter registry study with similar objectives. Specifically, the ALLIANCE A011202 trial (14) is randomizing patients with cT1–3cN1 breast cancer who have residual axillary disease post-NAT and surgical axillary staging to either ALND with radiation therapy (RT) to the undissected parts of the axilla and regional lymph nodes, or RT to all regional lymph nodes. Additionally, the ATNEC trial is comparing ALND or axillary RT with no further axillary treatment post-surgery in patients with early-stage (T1–3 N1 M0) breast cancer. Similarly, the TAXIS trial (15) is evaluating tailored axillary surgery followed by ALND and RT versus tailored axillary surgery followed by RT only. These studies, alongside our findings, are contributing to a robust body of evidence that supports surgical de-escalation in carefully selected patients to diminish the morbidity associated with more extensive axillary surgeries.

Another important consideration is the criteria of additional axillary interventions done in this study, such as ALND. From the patient characteristics in our study, the initial T stage before NAC was significantly higher in the SNB + ALND group compared to the SNB only group. Additionally, while not statistically significant, the histologic grade was higher in the SNB + ALND group, indicating more aggressive cancer features in this cohort. The SNB + ALND group also exhibited a greater proportion of hormone receptor-positive breast cancer, which typically shows less responsiveness to chemotherapy compared to HER2-positive or triple-negative breast cancer types. As seen in the comparison of characteristics between two groups, it is believed that these differences may have influenced the operators’ choice of axillary procedures. As a solution, we implemented PSM and even after matching, there were no significant differences in outcomes between the SNB only group and ALND adding group, which is considered a noteworthy result. In particular, the status of internal mammary lymph nodes (IMLN) and supraclavicular lymph nodes (SCLN) may influence decisions regarding the need for ALND in these patients. In this study, 180 patients had IMLN metastasis and 98 patients had SCLN metastasis. Of these, surgical dissection was performed on 4 out of the 180 IMLN cases and 13 out of the 98 SCLN cases after neoadjuvant chemotherapy. For clinical node stage 2 and 3 breast cancer, metastasis to IMLN or SCLN are included. One might think that the presence of IMLN or SCLN metastasis would need complete nodal clearance for more accurate staging. However, our subanalysis showed that, both before and after PSM, there was no significant difference in outcomes between the SNB only and SNB plus ALND groups, regardless of IMLN or SCLN involvement (**Supplementary Figure**).

To further improve the accuracy and safety of SNB, techniques such as targeted axillary dissection (TAD) and the dual-tracer method can be employed. TAD combines SNB with the removal of previously biopsied positive nodes, thereby reducing the risk of leaving behind metastatic disease. Recently, Sherko et al. conducted an assessment of 3-year outcomes in patients with node-positive breast cancer who underwent Targeted Axillary Dissection (TAD) alone versus those who underwent TAD in conjunction with Axillary Lymph Node Dissection (ALND). Their findings suggest that TAD alone may provide comparable survival outcomes and recurrence rates to those observed in patients receiving TAD + ALND (16). The dual method, which involves the use of both radioisotope and blue dye, improves the detection of sentinel lymph nodes and enhances the reliability of the procedure. These strategies have been shown to reduce the false-negative rate and may be particularly useful in patients with advanced nodal disease following NAC. Another study conducted by Ariane et al. introduces the MARI-protocol, which employs radioactive iodine seed marking to enhance the accuracy of sentinel lymph node biopsy (SNB). Their findings indicate positive outcomes, with the method preventing the need for axillary lymph node dissection (ALND) in 80% of clinically node-positive patients. Additionally, this approach demonstrated an excellent 3-year axillary recurrence-free interval, achieving a rate of 98% (17).

The limitations of this study include its single-center nature and retrospective design. Additionally, while clinical node stages 2 and 3 may involve metastasis to the IMLN and SCLN, there is a lack of detailed diagnostic and follow-up data, as well as a consensus on treatment approaches for these nodes. Furthermore, there may be discrepancies in the surgical decisions made by different operators regarding the necessity for further axillary surgery for each patient.

However, this study also possesses notable strengths. It is the first retrospective analysis to explore the omission of axillary lymph node dissection (ALND) in patients with clinical node stage 2 and 3 breast cancer taking into account the presence of metastasis in the IMLN and SCLN. As such, it provides valuable insights that contribute to the ongoing discourse on the de-escalation of axillary surgery, complementing other studies on this topic.

## Conclusion

In conclusion, our study shows that SNB alone may be sufficient for patients with clinical node stage 2 and 3 after NAC, as terms of ARFS, RRFS, OS compared to SNB + ALND. These findings support the potential for surgical de-escalation in axillary surgery without compromising oncologic outcomes, aligning with current trends in minimizing surgical aggressiveness to improve patient quality of life while maintaining effective treatment.

## Data Availability

The datasets analyzed during the current study are not publicly available as the personal information of patients must be protected but are available from the corresponding author on reasonable request. Requests to access these datasets should be directed to Eunju Shin, hellokkae@gmail.com.
