# Supplementary material for: Evaluating the survival outcomes in clinical node stage 2 and 3 breast cancer patients with negative sentinel lymph node biopsy after neoadjuvant chemotherapy: sentinel lymph node biopsy alone vs. axillary lymph node dissection
Source: Front Oncol. 2025 May 20;15:1563586. doi: 10.3389/fonc.2025.1563586 (PMC12129956; doi:10.3389/fonc.2025.1563586)
Supplement: Supplementary file 1 [file Presentation1.pptx]

## Slide 1
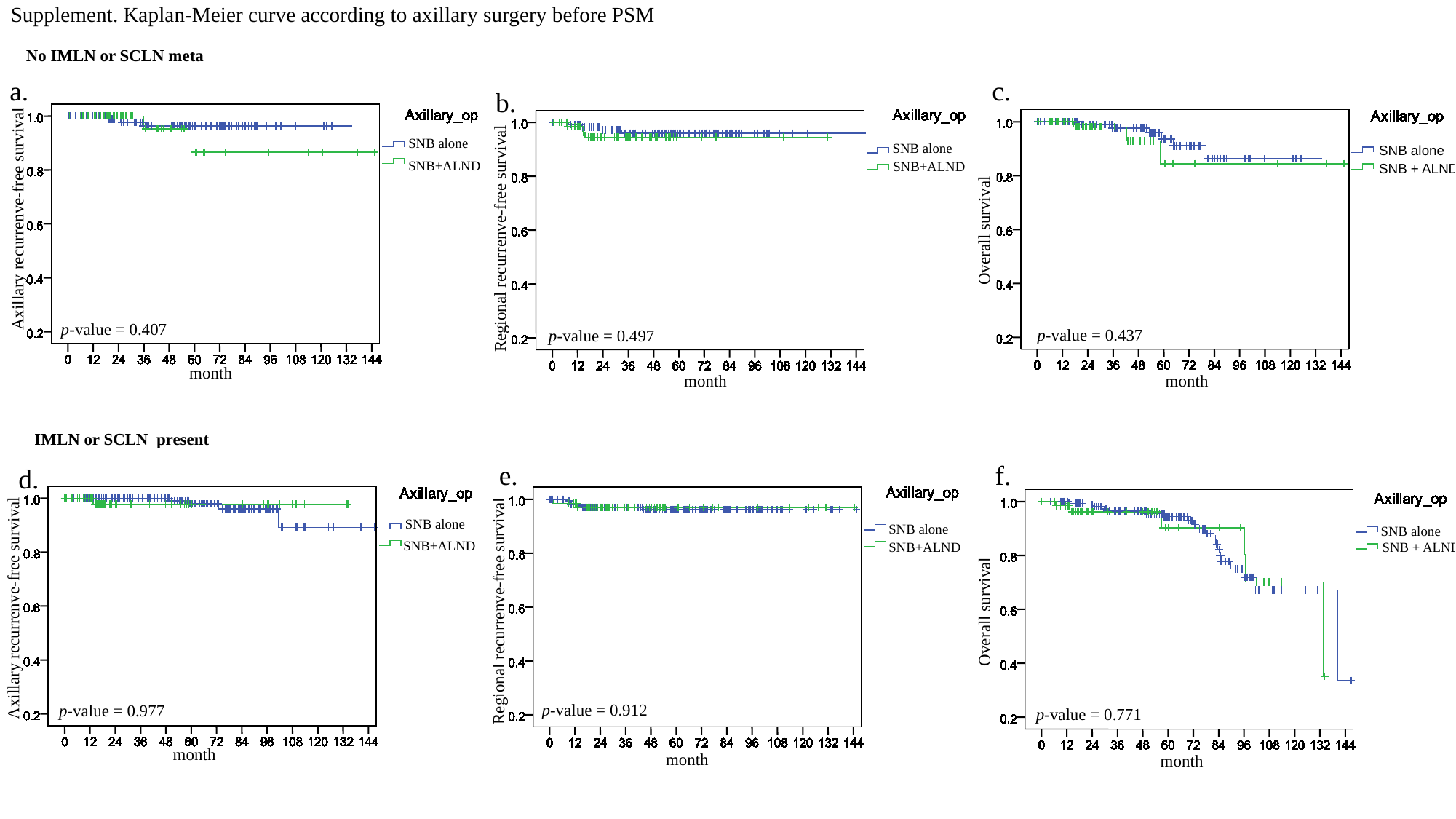

Supplement. Kaplan-Meier curve according to axillary surgery before PSM
No IMLN or SCLN meta
c.
a.
b.
SNB alone
SNB alone
SNB alone
SNB+ALND
SNB+ALND
SNB + ALND
Overall survival
Overall survival
Axillary recurrenve-free survival
Regional recurrenve-free survival
p-value = 0.407
p-value = 0.437
p-value = 0.497
month
month
month
 IMLN or SCLN present
e.
f.
d.
SNB alone
SNB alone
SNB alone
SNB+ALND
SNB + ALND
SNB+ALND
Overall survival
Axillary recurrenve-free survival
Regional recurrenve-free survival
p-value = 0.912
p-value = 0.977
p-value = 0.771
month
month
month

## Slide 2
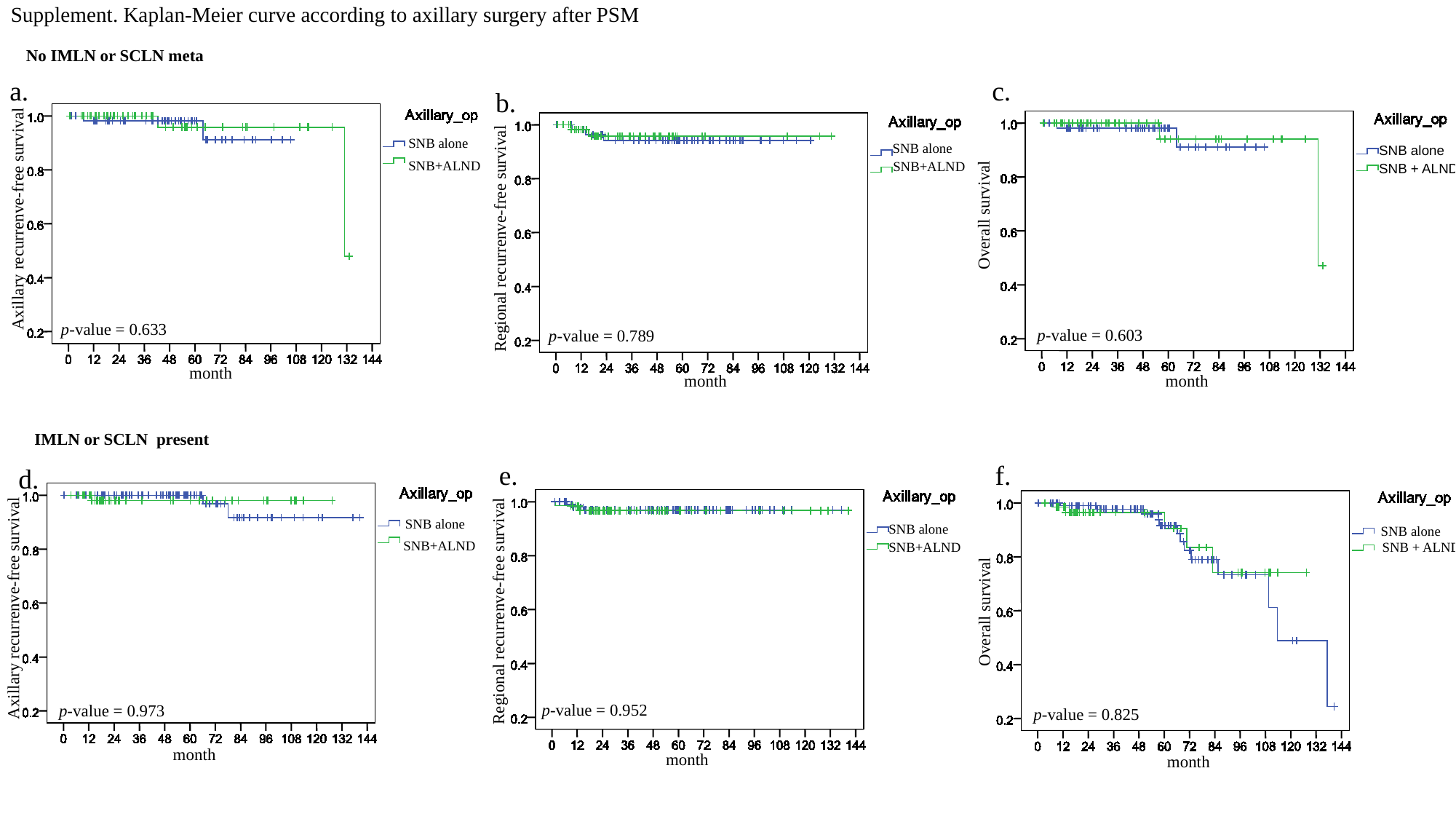

Supplement. Kaplan-Meier curve according to axillary surgery after PSM
No IMLN or SCLN meta
c.
a.
b.
SNB alone
SNB alone
SNB alone
SNB+ALND
SNB+ALND
SNB + ALND
Overall survival
Axillary recurrenve-free survival
Regional recurrenve-free survival
p-value = 0.633
p-value = 0.603
p-value = 0.789
month
month
month
 IMLN or SCLN present
e.
f.
d.
SNB alone
SNB alone
SNB alone
SNB+ALND
SNB + ALND
SNB+ALND
Overall survival
Axillary recurrenve-free survival
Regional recurrenve-free survival
p-value = 0.952
p-value = 0.973
p-value = 0.825
month
month
month
